# The predictive capacity of biomarkers for clinical pulmonary oedema in patients with severe falciparum malaria is low: a prospective observational study

**DOI:** 10.1186/s12936-024-05142-3

**Published:** 2024-10-24

**Authors:** Haruhiko Ishioka, Aniruddha Ghose, Hugh W. Kingston, Katherine Plewes, Stije J. Leopold, Ketsanee Srinamon, Prakaykaew Charunwatthana, Maswood Ahmed, A. K. M. Shamsul Alam, Anita Tuip-de Boer, Md Amir Hossain, Arjen M. Dondorp, Marcus J. Schultz

**Affiliations:** 1 Mahidol Oxford Tropical Medicine Research Unit, Faculty of Tropical Medicine, Mahidol University, Bangkok, Thailand; 2 Division of Infectious Diseases, Jichi Medical University Hospital, 3311-1 Yakushiji Shimotsuke-shi, Tochigi, 329-0498 Japan; 3 Chittagong Medical College Hospital, Chattogram, Bangladesh; 4 Department of Clinical Tropical Medicine, Faculty of Tropical Medicine, Mahidol University, Bangkok, Thailand; 5 Department of Intensive Care, Amsterdam University Medical Center, Amsterdam, Netherlands; 6 Centre for Tropical Medicine and Global Health, Nuffield Department of Clinical Medicine, University of Oxford, Oxford, UK

**Keywords:** Severe malaria, *Plasmodium falciparum*, Pulmonary oedema, Interleukin-6, Interleukin-8, Tumour necrosis factor, Soluble receptor of advanced glycation end-products, Surfactant protein-D, Club cell secretory protein, Krebs von den Lungen-6

## Abstract

**Background:**

Pulmonary oedema is a feared and difficult to predict complication of severe malaria that can emerge after start of antimalarial treatment. Proinflammatory mediators are thought to play a central role in its pathogenesis.

**Methods:**

An exploratory study was conducted to evaluate the predictive capacity of biomarkers for development of clinical pulmonary oedema in patients with severe falciparum malaria at two hospitals in Bangladesh. Plasma concentrations of interleukin-6 (IL-6), IL-8, tumour necrosis factor (TNF), soluble Receptor of Advanced Glycation End-products (sRAGE), surfactant protein-D (SP-D), club cell secretory protein (CC16), and Krebs von den Lungen-6 (KL-6) on admission were compared with healthy controls. Correlations between these biomarker and plasma lactate and *Plasmodium falciparum* histidine-rich protein 2 (PfHRP2) levels were evaluated. Receiver Operating Characteristic (ROC) curves were constructed to assess the predictive capacity for clinical pulmonary oedema of the biomarkers of interest.

**Results:**

Of 106 screened patients with falciparum malaria, 56 were classified as having severe malaria with a mortality rate of 29%. Nine (16%) patients developed clinical pulmonary oedema after admission. Plasma levels of the biomarkers of interest were higher in patients compared to healthy controls. IL-6, IL-8, TNF, sRAGE, and CC16 levels correlated well with plasma PfHRP2 levels (*r*_s_ = 0.39; *P* = 0.004, *r*_s_ = 0.43; *P* = 0.001, *r*_s_ = 0.54; *P* < 0.001, *r*_s_ = 0.44; *P* < 0.001, *r*_s_ = 0.43; *P* = 0.001, respectively). Furthermore, IL-6 and IL-8 levels correlated well with plasma lactate levels (*r*_s_ = 0.37; *P* = 0.005, *r*_s_ = 0.47; *P* < 0.001, respectively). None of the biomarkers of interest had predictive capacity for development of clinical pulmonary oedema.

**Conclusions:**

IL-6, IL-8, TNF, sRAGE, SP-D, CC16 and KL-6 cannot be used in predicting clinical pulmonary oedema in severe malaria patients.

**Supplementary Information:**

The online version contains supplementary material available at 10.1186/s12936-024-05142-3.

## Background

Malaria remains an important threat for global health in the era of post COVID-19 pandemic [1]. Pulmonary oedema is a well-known and severe complication carrying high mortality in patients with severe malaria [2]. Respiratory support such as mechanical ventilation is not always available in resource-limited settings where malaria is endemic, which increases mortality.

The pathogenesis of pulmonary oedema in severe malaria is not fully elucidated. Since hypoxaemia may manifest following the initiation of antimalarial therapy, it has been hypothesized that damage of endothelial cells in the lungs by proinflammatory cytokines increase pulmonary capillary permeability [3]. Vascular sequestration of parasitized red blood cells plays a central role in the multiorgan dysfunction in severe falciparum malaria [4], while the timing of the delayed onset questioned the contribution of sequestration on the complication of pulmonary oedema. Fluid overload could exacerbate pulmonary oedema; therefore, dry fluid management is required in patients with severe malaria [5, 6]. Intravenous fluid administration, however, is not a primary cause of the lung complications.

Predicting pulmonary oedema could be valuable in managing severe malaria by facilitating prompt respiratory support. In this exploratory study, therefore, the predictive capacity of various plasma biomarkers for clinical pulmonary oedema was assessed. In addition, the plasma levels of potential biomarkers in patients with malaria were compared to those in healthy volunteers, and it was determined whether they correlate with known prognostic factors, including plasma lactate and *Plasmodium falciparum* histidine-rich protein 2 (PfHRP2). The hypotheses were that the proinflammatory biomarkers interleukin (IL)-6, IL-8, tumour necrosis factor (TNF), and the lung injury biomarkers soluble Receptor of Advanced Glycation End-products (sRAGE), surfactant protein-D (SP-D), club cell secretory protein (CC16), and Krebs von den Lungen-6 (KL-6) have predictive capacity for the development of pulmonary oedema in patients with severe malaria. Selection of these biomarkers was based on the potential ability to predict pulmonary complications in diseases other than severe malaria reported in previous studies [7–10].

## Methods

### Study design

This was a prospective observational study, performed in the tertiary referral hospital Chittagong Medical College Hospital and in the subdistrict Ramu Upazila Health Complex in Bangladesh. Ethical approval for the study was obtained from the Ethical Review Committee of Chittagong Medical College and the Oxford Tropical Medicine Research Ethical Committee. Written informed consent was obtained from patients or attending relatives before enrollment. This study was registered at clinicaltrials.gov (identifier NCT01936766).

### Patients and clinical assessment

Consecutive patients admitted with falciparum malaria in the rainy seasons during 2013–2014 were included. Eligibility criteria for enrollment was presence of asexual *Plasmodium falciparum* parasitaemia confirmed by peripheral blood microscopy. The exclusion criteria was absence of consent.

The diagnosis of falciparum malaria was confirmed with microscopic examination of smears of peripheral blood specimens by Field stain. Severity of the disease was categorized according to modified World Health Organization (WHO) criteria [11], including coma (Glasgow coma scale < 11), clinical pulmonary oedema (SpO_2_ < 90% in ambient air with bilateral crepitation) [12], repeated convulsions, severe anemia or jaundice (hematocrit < 20% and bilirubin level of > 2.5 mg/dL, combined with parasite counts of > 100 000 parasites/µL), renal impairment (serum creatinine level of > 3 mg/dL and/or anuria), hypoglycaemia (blood glucose level of < 40 mg/dL), shock (systolic blood pressure of < 80 mm Hg with cool extremities), hyperparasitaemia (peripheral asexual stage parasitaemia level of > 10%), hyperlactataemia (venous plasma lactate level of > 4 mmol/L), or acidosis (venous plasma bicarbonate level of < 15 mmol/L). Uncomplicated malaria was defined as asexual *P. falciparum* slide positivity without severity criteria. Healthy individuals with no known acute or chronic illnesses were recruited locally as a comparison group for assessment of biomarker measurement. Acute respiratory distress syndrome (ARDS) was defined by the new global definition of acute respiratory distress syndrome [13].

A detailed medical history was recorded, and a physical examination was performed in all patients. Pulse oximetric saturation to fraction of inspired oxygen (SpO_2_/FiO_2_) ratio (S/F ratio) was calculated 24-hourly as an indicator for oxygenation instead of partial pressure of oxygen (PaO_2_)/FiO_2_ ratio (P/F ratio) [14]. The FiO_2_ values in low-flow oxygen-delivery system were predicted by oxygen flow rates described in Supplement 1 [15]. Fluid intake and urine output were recorded every 6 h. Antimalarial treatment was with intravenous artesunate, and standard supportive care was given according to WHO guidelines [16]. The availability of mechanical ventilation and renal replacement therapy was limited.

### Laboratory procedures

On admission, venous blood samples were taken for full blood count and routine biochemistry analysis. Parasitaemia was assessed 6-hourly from thick and thin smears until parasite clearance. Venous blood gas analysis and plasma lactate measurements were performed every 6 h until normalization (< 2 mmol/L) and then daily, using a handheld automated analyzer (i-STAT; Abbott). Arterial blood gas analysis was not always performed since the procedure was not a routine clinical practice in the study sites. Concentrations of PfHRP2 were measured using an enzyme-linked immunosorbent assay (Cellabs), as described before [17]. Plasma samples for biomarkers were shipped to and analysed in batches in the Amsterdam University Medical Centers, location AMC, the Netherlands. Levels of IL-6, IL-8, TNF, sRAGE, SP-D, CC16, and KL-6 in plasma were measured batchwise using customized Human Premixed Multi-Analyte Kit, Magnetic Luminex Screening Assay (R&D Systems, Minneapolis, Minnesota, USA), according to the manufacturer’s protocol as reported previously [18].

### Endpoint

The endpoint was the development of clinical pulmonary oedema after admission. Since radiological examination was not always available in malaria-endemic area due to the limitation of medical resources, clinical pulmonary oedema was defined as bilateral crepitation on auscultation combined with an oxygen saturation less than 90% on ambient air or equivalent saturations corrected for supplemented oxygen.

### Statistical analysis

A formal sample-size calculation was not performed. The number of consecutive patients in the rainy season served as the sample size. Differences in baseline demographic characteristics were compared using Mann–Whitney U tests (two groups) or Kruskal–Wallis tests (more than two groups) for continuous variables and the Fisher’s exact test for categorical variables. Levels of biomarkers of interest in malaria patients and in healthy volunteers were compared. Then, the correlations between the biomarkers of interest with the plasma lactate and PfHRP2 levels were assessed using the Spearman correlation coefficient.

Receiver operating characteristic (ROC) curve was constructed, and sensitivity and specificity, and negative and positive predictive value of each biomarker of interest were calculated to determine its predictive capability. The following cut-offs for the area under the ROC Curve (ROC–AUC) were used to classify the predictive value of biomarkers: 0.90 to 1.00 = excellent; 0.80 to 0.90 = good; 0.70 to 0.80 = fair; 0.60 to 0.70 = poor; and 0.50 to 0.60 = very poor. The optimal cut-off was determined using a Youden index for the selection of the highest sum of sensitivity and specificity. A 2-sided *P* value of < 0.05 was considered statistically significant. All analyses were performed using R Statistical Software version 4.2.3 (The R Foundation, Vienna, Austria).

## Results

### Demographics and baseline characteristics

A total of 106 patients with falciparum malaria were screened, of which 56 had severe malaria (Tables 1 and 2). Patients with falciparum malaria were 16 years of age or older except one 13-year-old girl with severe malaria included. Case fatality rate in severe malaria was 28.6%, and six patients died within 24 h of enrollment. Two patients with severe malaria had clinical pulmonary oedema at admission. Nine of 54 (16.7%) newly developed clinical pulmonary oedema. Eight of nine patients (88.9%) were women. Among five pregnant women included in the study, three developed pulmonary oedema after admission. The median time from enrollment to the development of clinical pulmonary oedema was 28 (IQR, 13–31) hours, and 4 of 9 (44.4%) developed it within 24 h. According to the new global definition of ARDS definition, both patients with clinical pulmonary oedema at admission had ARDS, and seven out of nine patients who developed pulmonary oedema post-admission had ARDS. Longitudinal changes of S/F ratio in patients with severe malaria stratified by the development of clinical pulmonary oedema is shown in Table 3. There were no significant differences in the total fluid intake and cumulative fluid balance between patients who developed clinical pulmonary oedema after admission and those who did not (Table 4).

**Table 1:** Baseline characteristics of patients with severe and uncomplicated malaria, and healthy individuals

Variables	n	Severe malaria (*n* = 56)	n	Uncomplicated malaria (*n* = 50)	n	Healthy individuals (*n* = 16)	*P* value	*P* value
							Overall	SM vs. UM
Age, year	56	28 [22 to 41]	50	30 [22 to 42]	16	28 [26 to 34]	0.977	0.872
Male gender, n (%)	56	37 (66.1)	50	34 (68.0)	16	15 (93.8)	0.084	1.000
Pregnancy, n (%)	56	5 (8.9)	50	2 (4.0)	16	0 (0.0)	0.315	0.530
Clinical measures								
Temperature, °C	56	38.2 [37.5 to 39.1]	50	37.8 [37.2 to 39.3]	16	36.8 [36.6 to 37.0]	< 0.001	0.506
Respiratory rate, /min	56	34 [28 to 43]	50	27 [22 to 32]	16	17 [14 to 23]	< 0.001	< 0.001
Pulse rate, /min	56	114 [101 to 125]	50	102 [89 to 113]	16	75 [70 to 82]	< 0.001	0.002
Systolic blood pressure, mmHg	56	110 [100 to 118]	50	107 [100 to 114]	16	127 [121 to 135]	< 0.001	0.293
Mean blood pressure, mmHg	55	80 [71 to 88]	50	80 [69 to 86]	16	94 [86 to 97]	0.001	0.424
Glasgow coma scale	56	9 [8 to 15]	50	15 [15 to 15]	16	15 [15 to 15]	< 0.001	< 0.001
Laboratory measures								
Haemoglobin, g/dL	56	9.1 [7.1 to 10.9]	50	11.0 [9.0 to 13.2]	16	14.1 [13.1 to 15.1]	< 0.001	< 0.001
Creatinine, mg/dL	56	1.7 [1.2 to 3.3]	49	0.9 [0.8 to 1.2]	16	0.9 [0.9 to 1.0]	< 0.001	< 0.001
Base deficit, mEq/L	56	8 [5 to 11]	50	3 [1 to 5]	16	− 2 [− 2 to − 1]	< 0.001	< 0.001
Lactate, mmol/L	56	3.9 [2.4 to 5.9]	50	1.6 [1.2 to 2.1]	16	1.1 [0.9 to 1.4]	< 0.001	< 0.001
Parasitological measures								
Parasitaemia, /µL	56	119,006 [16532 to 355398]	50	17,521 [1360 to 99915]		NA	NA	< 0.001
PfHRP2, ng/mL	54	3559 [1759 to 10219]	49	563 [128 to 1221]		NA	NA	< 0.001

All data as median [IQR], except where otherwise indicated

The number of cases (n) in each group are 56, 50, and 16, respectively, except where indicated

Overall *P* value is from Kruskal–Wallis test comparing groups of severe malaria, uncomplicated malaria, and healthy individuals

*SM* severe malaria, *UM* uncomplicated malaria, *PfHRP2* *Plasmodium falciparum* histidine rich protein 2, *NA* not applicable

**Table 2:** Baseline characteristics of patients with severe falciparum malaria, stratified by the development of clinical pulmonary oedema after admission

Variables	n	Non-CPE patients (*n* = 45)	n	CPE patients^a^ (*n* = 9)	*P* value
Age, year	45	28 [22 to 45]	9	32 [24 to 35]	0.789
Male gender, n (%)	45	34 (75.6)	9	1 (11.1)	0.001
Pregnancy, n (%)	45	2 (4.4)	9	3 (33.3)	0.028
Clinical measures					
Temperature, °C	45	38.2 [37.5 to 39.2]	9	37.6 [36.8 to 38.7]	0.218
Respiratory rate, /min	45	32 [28 to 38]	9	44 [40 to 47]	0.023
Pulse rate, /min	45	110 [98 to 124]	9	119 [112 to 124]	0.171
Systolic blood pressure, mmHg	45	113 [100 to 119]	9	110 [100 to 117]	0.719
Mean blood pressure, mmHg	44	82 [73 to 88]	9	73 [71 to 83]	0.507
Glasgow coma scale	45	9 [8 to 15]	9	9 [9 to 14]	0.953
Laboratory measures					
Haemoglobin, g/dL	45	9.4 [7.1 to 10.9]	9	7.2 [5.6 to 8.3]	0.024
Creatinine, mg/dL	45	1.6 [1.2 to 3.2]	9	2.0 [1.3 to 3.7]	0.609
Base deficit, mEq/L	45	8 [5 to 10]	9	11 [8 to 16]	0.025
Lactate, mmol/L	45	3.3 [2.3 to 5.1]	9	4.0 [3.3 to 9.1]	0.270
Parasitological measures					
Parasitaemia, /µL	45	114,924 [18840 to 360522]	9	212,854 [51094 to 353690]	0.562
PfHRP2, ng/mL	43	3642 [1808 to 10128]	9	3015 [2272 to 6194]	0.971
Severity of diseases					
Cerebral malaria, GCS < 11, n (%)	45	27 (60.0)	9	5 (55.6)	1.000
Renal impairment, creatinine ≥ 3.0 mg/dL, n (%)	45	16 (35.6)	9	4 (44.4)	0.712
Hypotension, SBP < 80 mmHg, n (%)	45	1 (2.2)	9	0 (0.0)	1.000
Metabolic acidosis, HCO_3_ < 15 mmol/L, n (%)	45	12 (26.7)	9	5 (55.6)	0.121
Hyperlactataemia, lactate ≥ 4 mmol/L, n (%)	45	21 (46.7)	9	5 (55.6)	0.724
Mechanical ventilation, n (%)	45	4 (8.9)	9	3 (33.3)	0.081
Blood transfusion, n (%)	45	19 (42.2)	9	7 (77.8)	0.072
Renal replacement therapy, n (%)	45	14 (31.1)	9	6 (66.7)	0.062
Fatal outcome, n (%)	45	10 (22.2)	9	5 (55.6)	0.096

All data as median [IQR], except where otherwise indicated

The number of cases (n) in each group are 45 and 9, respectively, except where indicated

*CPE* clinical pulmonary oedema, *PfHRP2* *Plasmodium falciparum* histidine rich protein 2, *GCS* Glasgow coma scale, *SBP* systolic blood pressure; *HCO*_*3*_ bicarbonate

^a^This group includes patients with severe malaria who developed clinical pulmonary oedema after admission. Two patients who had clinical pulmonary oedema on admission are not included in this group

**Table 3:** SpO_2_/FiO_2_ ratio among patients with severe malaria, stratified by development of clinical pulmonary oedema after admission

Variables	*n*	Non-CPE patients (*n* = 45)	*n*	CPE patients^a^ (*n* = 9)	*P* value
SpO_2_/FiO_2_ ratio at admission	45	452 [443 to 462]	9	452 [438 to 462]	0.833
SpO_2_/FiO_2_ ratio at 24-h timepoint	39	452 [446 to 462]	9	272 [157 to 443]	0.008
SpO_2_/FiO_2_ ratio at 48-h timepoint	37	457 [452 to 462]	6	244 [182 to 376]	0.002

All data as median [IQR]

SpO_2_/FiO_2_ ratio of two patients who had clinical pulmonary oedema on admission were 336 and 424, respectively

*CPE* clinical pulmonary oedema

^a^This group includes patients with severe malaria who developed clinical pulmonary oedema after admission. Two patients who had clinical pulmonary oedema on admission are not included in this group

**Table 4:** Fluid intake and cumulative fluid balance among patients with severe malaria, stratified by development of clinical pulmonary oedema after admission

Variables	Non-CPE patients (*n* = 45)					CPE patients^a^ (*n* = 9)					*P* value	*P* value
	n	mL		mL/kg/h		n	mL		mL/kg/h		mL	mL/kg/h
First 6 h from enrollment												
Intravenous fluid administered	44	810	[440 to 1530]	2.6	[1.4 to 4.7]	9	1000	[550 to 1100]	3.0	[1.8 to 3.5]	0.785	0.972
Total fluid intake	44	1000	[500 to 1710]	2.9	[1.6 to 5.9]	9	1000	[550 to 1180]	3.0	[1.8 to 3.5]	0.991	0.776
Fluid balance	43	600	[250 to 1380]			9	870	[450 to 1060]			0.570	NA
First 24 h from enrollment												
Intravenous fluid administered	40	2370	[1500 to 3140]	2.0	[1.1 to 3.0]	9	2220	[1650 to 2650]	1.7	[1.1 to 2.2]	0.969	0.588
Total fluid intake	39	2660	[1800 to 3730]	2.2	[1.3 to 3.2]	9	2500	[1650 to 2750]	1.9	[1.1 to 2.3]	0.771	0.391
Fluid balance	34	1350	[660 to 2500]			9	2000	[710 to 2470]			0.765	NA

All data as median [IQR]

*CPE* clinical pulmonary oedema, *NA* not applicable

^a^This group includes patients with severe malaria who developed clinical pulmonary oedema after admission. Two patients who had clinical pulmonary oedema on admission are not included in this group

### Biomarker levels on admission

The plasma concentrations of IL-6, IL-8, and TNF were increased in proportion to disease severity among three groups of patients with severe malaria, uncomplicated malaria, and healthy individuals, while the biomarkers for lung injury did not show such a trend (Table 5). Levels of IL-6, IL-8, TNF, sRAGE, and CC16 were positively correlated with PfHRP2 (Table 6A). The IL-6 and IL-8 levels were positively correlated with plasma lactate on admission (Table 6B).

**Table 5:** Biomarkers at admission among patients with falciparum malaria and healthy individuals

Variables	n	Severe malaria					n	Uncomplicated malaria (*n* = 50)	n	Healthy individuals (*n* = 16)	*P* value^a^
		Non-CPE patients (*n* = 45)	n	CPE patients^b^ (*n* = 9)	n	All^c^ (*n* = 56)					
IL-6, pg/mL	45	192 [89 to 521]	9	287 [77 to 767]	56	193 [87 to 732]	50	53 [15 to 148]	16	2 [2 to 3]	0.880
IL-8, pg/mL	45	65 [32 to 107]	9	190 [60 to 282]	56	66 [31 to 172]	48	23 [13 to 47]	11	10 [6 to 12]	0.232
TNF, pg/mL	45	144 [70 to 222]	9	135 [86 to 241]	56	140 [71 to 245]	49	54 [27 to 77]	11	7 [4 to 30]	0.736
RAGE, pg/mL	45	4312 [2744 to 7509]	9	4569 [3330 to 6496]	56	4514 [2744 to 7345]	50	2603 [2180 to 3332]	16	2585 [2168 to 3149]	0.991
SP-D, pg/mL	45	3323 [962 to 5644]	9	2766 [1478 to 6129]	56	3252 [1313 to 5971]	48	2865 [1427 to 6900]	16	7321 [5740 to 9934]	0.790
CC-16, pg/mL	45	11,299 [7266 to 21128]	9	8796 [7133 to 26947]	56	11,471 [7233 to 21572]	50	6460 [3720 to 10081]	16	12,289 [9445 to 15698]	0.790
KL-6, U/mL	45	17 [10 to 25]	8	21 [14 to 47]	55	18 [10 to 28]	49	18 [12 to 23]	16	24 [17 to 29]	0.253

All data as median [IQR]

The number of patients (n) in each group are 45, 9, 56, 50, and 16, respectively, except where indicated

*CPE* clinical pulmonary oedema, *IL-6* interleukin-6, *IL-8* interleukin-8, *TNF* tumour necrosing factor, *sRAGE* soluble receptor of advanced glycation end-products, *SP-D* surfactant protein D, *CC16* club cell secretory protein, *KL-6* Krebs von den Lungen 6

^a^For comparison between non-CPE patients vs. CPE patients with severe malaria

^b^This group includes patients with severe malaria who developed clinical pulmonary oedema after admission. Two patients who had clinical pulmonary oedema on admission are not included in this group

^c^This group includes two patients who had clinical pulmonary oedema on admission

**Table 6:** Correlation between prognostic factors (PfHRP2 and lactate) and biomarkers at admission among patients with severe malaria

**A. Plasma PfHRP2 and biomarkers on admission**		
Variables	Spearman’s coefficient (*r*_s_)	Significance
IL-6	0.39	0.004
IL-8	0.43	0.001
TNF	0.54	< 0.001
sRAGE	0.44	< 0.001
SP-D	− 0.10	0.465
CC16	0.43	0.001
KL-6	0.15	0.274
**B. Plasma lactate and biomarkers on admission**		
Variables	Spearman’s coefficient (*r*_s_)	Significance
IL-6	0.37	0.005
IL-8	0.47	< 0.001
TNF	0.22	0.097
sRAGE	− 0.06	0.684
SP-D	− 0.25	0.064
CC16	− 0.21	0.119
KL-6	0.01	0.934

*PfHRP2* *Plasmodium falciparum* histidine-rich protein 2, *IL-6* interleukin-6, *IL-8* interleukin-8, *TNF* tumour necrosing factor, *sRAGE* soluble receptor of advanced glycation end-products, *SP-D* surfactant protein D, *CC16* club cell secretory protein, *KL-6* Krebs von den Lungen 6

### Predictive value of the tested biomarkers in severe malaria

**Fig. 1:**
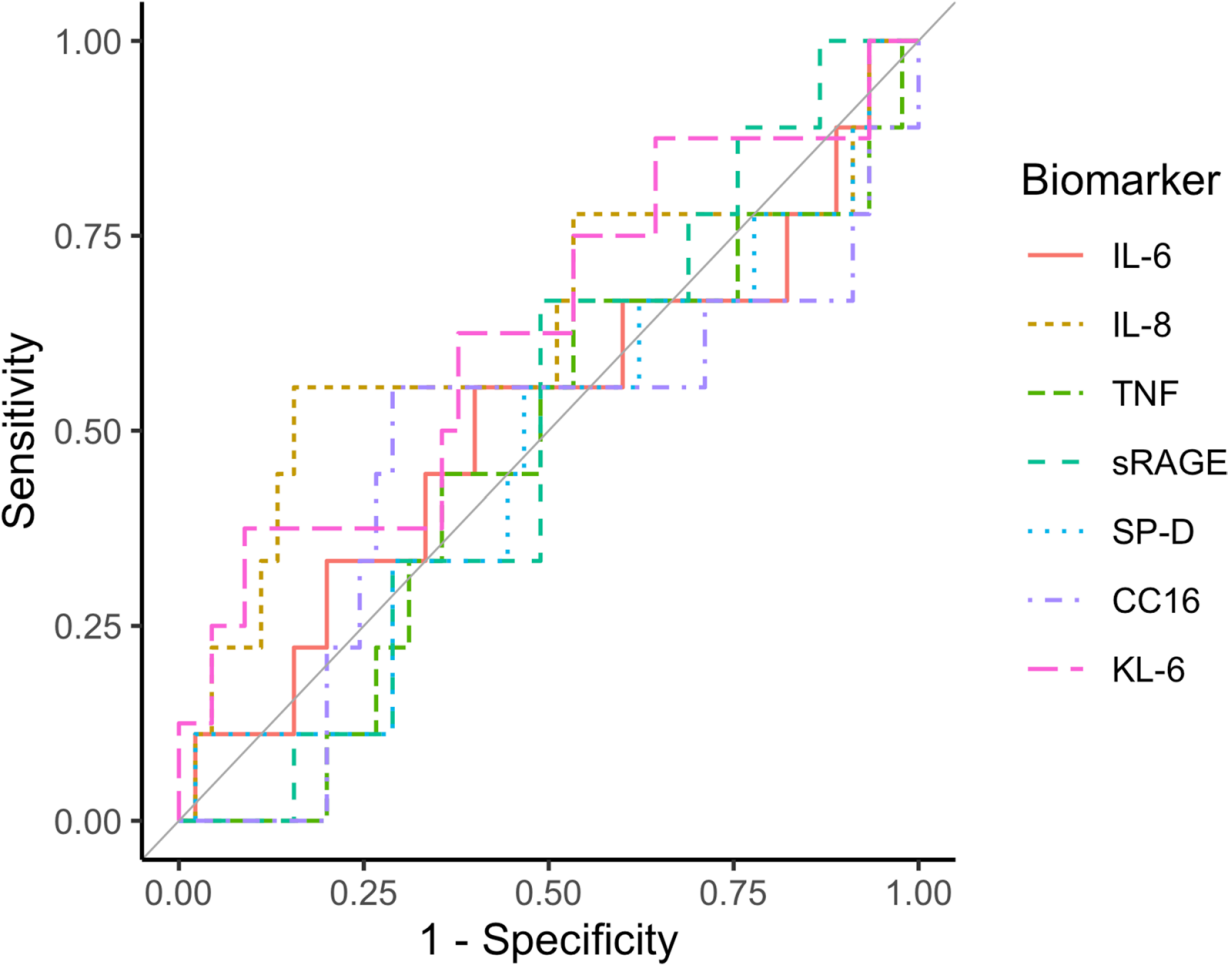
Receiver operating characteristic curves of IL-6, IL-8, TNF, sRAGE, SP-D, CC16, and KL-6 for the prediction of clinical pulmonary oedema in severe malaria

The levels of all biomarkers in patients developed clinical pulmonary oedema after admission were not significantly different from those in patients who did not (Table 5). Two patients complicated with clinical pulmonary oedema on admission were excluded from the analysis. ROC curves are shown in Fig. 1. The diagnostic accuracy of all biomarkers was low (Table 7).

**Table 7:** AUC, sensitivity, specificity, negative predictive value, positive predictive value, and cut-off value of the biomarkers

Variables	AUC	95% CI	Sensitivity (%)	Specificity (%)	NPV (%)	PPV (%)	Cut-off value
IL-6	0.52	0.28 to 0.75	56	60	87	22	> 282 pg/mL
IL-8	0.63	0.38 to 0.87	56	84	90	42	> 184 pg/mL
TNF	0.46	0.25 to 0.67	67	47	88	20	< 148 pg/mL
sRAGE	0.50	0.31 to 0.68	67	51	88	21	> 4386 pg/mL
SP-D	0.47	0.25 to 0.69	56	53	86	19	< 2974 pg/mL
CC16	0.47	0.23 to 0.71	56	71	89	28	< 9114 pg/mL
KL-6	0.63	0.39 to 0.87	38	91	89	43	> 41.5 U/mL

The analysis was based on patients with severe malaria, excluding two patients who developed clinical pulmonary oedema on admission

*AUC* area under the curve, *CI* confidence interval, *NPV* negative predictive value, *PPV* positive predictive value, *IL-6* interleukin-6, *IL-8* interleukin-8, *TNF* tumour necrosing factor, *sRAGE* soluble receptor of advanced glycation end-products, *SP-D* surfactant protein D, *CC16* club cell secretory protein, *KL-6* Krebs von den Lungen 6

## Discussion

The primary objective of this study was to determine the predictive value of admission biomarkers for the development of pulmonary oedema in patients with severe malaria. Contrary to the hypotheses, a poor predictive value for pulmonary oedema of the biomarkers of interest were found. The proinflammatory biomarkers were increased in proportion to disease severity, while no such trend was observed for the lung injury biomarkers. There were significant correlations between proinflammatory biomarkers and PfHRP2, similar to IL-6 or IL-8 and plasma lactate, whereas sRAGE and CC16 were the only lung injury markers which showed correlations with PfHRP2.

This study had several strengths of a prospective nature with a clearly defined study population of high severity, at high risk of pulmonary oedema. Patients with clinical pulmonary oedema on admission were excluded from the analysis for biomarker predictivity. A large set of biomarkers that have shown to have associations with outcome in previous studies was used. The batchwise measurements was advantageous method to prevent laboratory variations.

Neutrophils are suggested to play an essential role in the pathogenesis of ARDS [19], and thus probably also in patients that do not meet the definition of ARDS, i.e., critically ill patients with acute hypoxaemic respiratory failure. Activated neutrophils migrate from the bloodstream into the lung tissues, releasing cytokines, proteases, and reactive oxygen species (ROS) and generate neutrophil extracellular traps (NETs), which can contribute to lung injury and consequently pulmonary oedema. In this study, however, the plasma concentrations of IL-6, IL-8, and TNF, which can induce the activation of neutrophils, were not associated with the development of clinically pulmonary oedema in severe malaria, suggesting that severe malaria might have a specific pathophysiology for the development of pulmonary oedema different from the estimated mechanisms in ARDS from other causes, such as a re-perfusion damage after the release of sequestration.

In line with previous investigations, biomarkers for inflammation were rather correlated with prognostic indicators of plasma PfHRP2. Indeed, a correlation between IL-6 and plasma lactate was found in a previous study [20]. These results implied that inflammatory cytokines of IL-6, IL-8, and TNF may be associated with the sequestration and consequent endothelial dysfunction, the primary pathogenesis of severe malaria. Important in this context is that there is no evidence that a higher parasite biomass is more likely to cause pulmonary oedema.

Interestingly, biomarkers for lung injury did not correlate with known prognostic predictors and were also not predictive of pulmonary oedema. RAGE is a member of the immunoglobulin super-family and a multiligand transmembrane receptor expressed mainly in the alveolar type-1 cells. sRAGE, the form lacking the transmembrane domain, might be a candidate predictor for the diagnosis of ARDS [7, 8, 21]. The concentrations of sRAGE were correlated with parasite biomass but did not predict clinical pulmonary oedema from the ROC curves. Since the levels of plasma CC16, a small 16 kDa protein secreted by the club cells, in patients with severe malaria was similar with those in healthy individuals in this study, its clinical significance for lung injury was unclear in severe malaria. Previous studies reported that the lower concentrations of CC16 have been reported to be associated with the diagnosis of ARDS [7, 22], while the other observational study showed the opposite association of its higher concentrations with ARDS diagnosis [23].

One plausible explanation for the lack of correlation between the biomarkers and the development of clinical pulmonary oedema was the temporal dynamics of mediator responses. In patients with severe malaria, pulmonary oedema often develops after initiation of antimalarial drugs. The median time from hospitalization to the onset of pulmonary oedema in this study was 30 h. In this study, sampling was performed on admission promptly after participant consent was obtained. For future research, it may be possible to capture markers related to the pathogenesis of pulmonary oedema by longitudinal studies with biomarker profiling over time after initiation of therapeutic agents, specifically during first 24 after hospitalization and start of therapy. However, substances whose plasma concentrations rapidly change over a short period of time have a narrow monitoring window and may not be suitable for clinical predictors.

This study had limitation of small sample size, but a recent decline of the number of patients with severe malaria makes further implementation of the research more challenging. Pulmonary oedema defined in this study had no radiological confirmation. The population with clinical pulmonary oedema may have included patients with conditions other than pulmonary oedema, though they were clinically differentiated from aspiration pneumonia. Among patients with severe malaria, six patients died within 24 h. One of them had clinical pulmonary oedema on admission but the rest of them did not develop pulmonary oedema, based on the collected data. It is not possible to definitively determine whether these patients actually progressed to pulmonary oedema. However, the biomarker levels in these five patients were not obviously high, and their inclusion in the group of patients with pulmonary oedema did not materially affect the ROC–AUC results in the additional analysis.

## Conclusions

The prognostic capacity of biomarkers, plasma IL-6, IL-8, TNF, sRAGE, SP-D and KL-6 on admission for a prediction of clinical pulmonary oedema in severe malaria is poor. Pro-inflammatory mediators were rather correlated with parasite biomass.

## Supplementary Information

Supplementary Material 1.

## Data Availability

No datasets were generated or analysed during the current study.

## Abbreviations

ARDS: Acute respiratory distress syndrome
CC16: Club cell secretory protein
FiO_2_: Fraction of inspired oxygen
IL-6: Interleukin-6
IL-8: Interleukin-8
KL-6: Krebs von den Lungen-6
PfHRP2: *Plasmodium falciparum* histidine-rich protein 2
ROC: Receiver operating characteristic
ROC–AUC: Area under the ROC Curve
SpO_2_: Pulse oximetric saturation
SP-D: Surfactant protein-D
sRAGE: Soluble receptor of advanced glycation end-products
TNF: Tumour necrosis factor
