# Supplementary material for: The predictive capacity of biomarkers for clinical pulmonary oedema in patients with severe falciparum malaria is low: a prospective observational study
Source: Malar J. 2024 Oct 24;23:320. doi: 10.1186/s12936-024-05142-3 (PMC11515577; doi:10.1186/s12936-024-05142-3)
Supplement: Supplementary file 1 — Supplementary Material 1. [file 12936_2024_5142_MOESM1_ESM.docx]

Supplement 1. Oxygen flow rates and FiO_2_.

| Low-flow system | Oxygen flow rates (L/min) | FiO_2_ |
| --- | --- | --- |
| Nasal cannula | 1 | 0.24 |
|  | 2 | 0.28 |
|  | 3 | 0.32 |
|  | 4 | 0.36 |
|  | 5 | 0.40 |
|  | 6 | 0.44 |
|  |  |  |
| Face mask | 5-6 | 0.40 |
|  | 6-7 | 0.50 |
|  | 7-8 | 0.60 |
|  |  |  |
| Nonrebreathing mask | 10 | 0.80 |
|  | 15 | 0.90 |

Modified from Table 11-2 in Shapiro BA, Peruzzi WT, Templin R. Clinical application of blood gases, 5th ed. Chicago: Mosby; 1994.
